# Improving health and the delivery of care using digital technologies: Special issue highlighting innovative approaches to incorporating digital technology into health care

**DOI:** 10.1016/j.pecinn.2023.100184

**Published:** 2023-06-22

**Authors:** Jordan M. Alpert

**Affiliations:** Cleveland Clinic, Internal Medicine and Geriatrics, USA

Innovation is the driving force behind health care improvement. Medical devices such as X-ray machines, MRIs, wearable monitors, and robotic surgery systems are examples of technological innovations. Informatics, such as personal health records, natural language processing, and conversational agents, are further examples of innovations. Such innovations can only succeed in practice if they suit the needs of users, outperform alternative methods, and are simple to understand [1].

The advancement of digital technology has accelerated the development of health care tools. More than 350,000 health and wellness applications, for example, are available for download, with as many as 90,000 new health apps created each year [2]. The COVID-19 pandemic has been linked to increased use of digital tools, which has altered patient-physician interactions. As a result, telehealth services skyrocketed from 2.1 million to 32.5 million in a sample of five U.S. states between 2020 and 2021 [3]. Furthermore, electronic health records were used more frequently—in a national study, clinicians received a 157% increase in patient messages compared to pre-pandemic levels [4]. Never before has digital technology had such an impact on health care.

This special issue of *Patient Education and Counseling Innovation* demonstrates aspects of digital technology that should be considered during the development, testing, and implementation phases. Topics covered in the 17 articles range from web-based video programs to text-message reminder systems that covered the continuity of care, from conceptual testing to the evaluation of full interventions. Obesity, neurofibromatosis, kidney disease, and cognitive impairment were among the many conditions included. Four intervention-related articles assessed patients' and providers' perceptions of proposed designs; three articles concentrated on mHealth educational interventions; and two articles evaluated interventions to improve the quality of care. The remaining eight articles encompassed telehealth, health literacy, and feasibility testing: two of the three telehealth articles examined telehealth usage from the provider's perspective, while the patient-focused article looked at engagement levels after using the system; two health literacy articles were about using eHealth tools to strengthen patients' literacy skills; two articles investigated the feasibility of tools to help patients manage a disease; and one article conducted a feasibility test for a health literacy application.

In addition to introducing innovative tools and promising interventions that can enhance the delivery of care, articles in this special issue also included novel methods. Specifically, Zwi and colleagues employed multi-site, pre-post, and mixed-methods to examine the feasibility of a cross-platform eHealth tool for patients with hemodialysis [5]**,** while Ayre and colleagues used an iterative approach to collect real-time, actionable feedback for a health literacy tool [6].

As digital tools become more prevalent in health care, this special issue can serve as a foundation for understanding all the various factors that should be considered to advance both theory and practice.
